# The Potential Role of Cytokine Storm Pathway in the Clinical Course of Viral Respiratory Pandemic

**DOI:** 10.3390/biomedicines9111688

**Published:** 2021-11-15

**Authors:** Giuseppe Murdaca, Francesca Paladin, Alessandro Tonacci, Stefania Isola, Alessandro Allegra, Sebastiano Gangemi

**Affiliations:** 1 Clinical Immunology Unit, Department of Internal Medicine, University of Genoa and Ospedale Policlinico San Martino, 16132 Genoa, Italy; 2 Department of Internal Medicine, University of Genoa and Ospedale Policlinico San Martino, 16132 Genoa, Italy; puell-a@hotmail.it; 3 Clinical Physiology Institute, National Research Council of Italy (IFC-CNR), 56124 Pisa, Italy; atonacci@ifc.cnr.it; 4 School and Operative Unit of Allergy and Clinical Immunology, Department of Clinical and Experimental Medicine, University of Messina, 98125 Messina, Italy; stefania.isola@unime.it (S.I.); sebastiano.gangemi@unime.it (S.G.); 5 Division of Hematology, Department of Human Pathology in Adulthood and Childhood “Gaetano Barresi”, University of Messina, 98125 Messina, Italy; aallegra@unime.it

**Keywords:** COVID-19, SARS, MERS, Spanish flu, H1N1, cytokine storm, hemostasis

## Abstract

The “cytokine storm” (CS) consists of a spectrum of different immune dysregulation disorders characterized by constitutional symptoms, systemic inflammation and multiorgan dysfunction triggered by an uncontrolled immune response. Particularly in respiratory virus infections, the cytokine storm plays a primary role in the pathogenesis of respiratory disease and the clinical outcome of respiratory diseases, leading to complications such as alveolar edema and hypoxia. In this review, we wanted to analyze the different pathogenetic mechanisms involved in the various respiratory viral pandemics (COVID-19; SARS; MERS; H1N1 influenza A and Spanish flu) which have affected humans in this and last century, with particular attention to the phenomenon of the “cytokine storm” which determines the clinical severity of the respiratory disease and consequently its lethality.

## 1. Introduction

Respiratory viral infection results in the activation and recognition by cytotoxic T lymphocytes, resulting in the overproduction of a wide range of proinflammatory mediators (IL-1β, IFN-γ, IP-10, monocyte chemotactic protein 1 (MCP-1) and IL-17), in addition to the activation of basophils and mast cells, causing microvascular losses and a procoagulative state. It is in this context that the “cytokine storm” syndrome develops, the main actor involved in the clinical severity of respiratory infection, up to acute respiratory distress syndrome (ARDS) [1]. This condition has also been described in the SARS-CoV-2 infection pandemic as a major factor worsening clinical outcomes in infected people. For this reason, the purpose of this study was to review the relevant scientific information regarding the potential role of the cytokine storm during the clinical course of major viral respiratory pandemics (SARS, MERS, SARS-CoV-2 and influenza). The results of this review highlight the importance of the cytokine storm during the infection of these pandemic viruses, suggesting that this condition must be considered as a relevant factor in the election of medical treatments for these viral diseases.

For this purpose, we have selected scientific publications on specific topics, “COVID-19, SARS, MERS, Spanish flu, H1N1 and cytokine storm”, from www.pubmed.gov accessed on 21 October 2021. The primary endpoint was to select research papers investigating the role of the cytokine storm in the pathogenesis and clinical severity of the different viral infections analyzed. By carefully reading the individual articles, we have identified the specific cytokines and their suggested role in the progression and the severity of the infection. Therefore, the secondary endpoint was to propose a possible connection point between the different types of viral diseases, and also in order to identify the most appropriate treatment capable of slowing down the progression of the more severe forms.

## 2. Results and Discussion

### 2.1. Cytokine Storm

The term “cytokine storm” (CS) refers to a spectrum of different immune dysregulation disorders characterized by constitutional symptoms, systemic inflammation and multiorgan dysfunction [2]. The trigger for the CS is an uncontrolled immune response with the continuous activation and expansion of immune cells, lymphocytes and macrophages, which produce immense amounts of proinflammatory cytokines [3]. In viral infections, the release of proinflammatory factors leads to apoptosis of lung epithelial cells, and pulmonary alveolar and microvascular endothelial cells, leading to complications such as alveolar edema and hypoxia. The uncontrolled production of proinflammatory factors, containing IL-6, IL-8, IL-1β and GM-CSF, and chemokines such as CCL2, CCL-5, IP-10 and CCL3, along with reactive oxygen species, results in pulmonary fibrosis [4]. CXCL8 chemokine is also involved in the inflammation and trafficking of immune cells in the context of viral infections, as it has a chemotactic action for neutrophils and monocytes in the respiratory tract [5]. The clinical picture of the cytokine storm, although variable, generally manifests itself as laboratory “overlap syndrome” with a decreased cell count, a decreased ESR, an increased ferritin level, NK dysfunction and hemophagocytosis [6]. This clinically results in the increased perivascular infiltration of activated macrophages, neutrophils and fibroblasts, accompanied by extensive fibrin deposition and alveolar collapse, leading to acute respiratory distress syndrome (ARDS) [7,8].

### 2.2. Cytokine Storm and Hemostasis

During inflammation, endothelial cells (ECs) are stimulated and generate tissue factors, which in turn trigger the extrinsic coagulation pathway, causing a hypercoagulable state [9]. The cytokine storm can also produce an endothelial impairment and dysfunction, the alteration of coagulation, the change of microvascular permeability, hemorrhages and thrombosis [10].

In fact, the responsibility of ECs is the preservation of the vascular task for blood fluidity, along with the avoidance of thromboembolic incidents. Thus, ECs have a double function in the coagulation system. This means that they play a part in the clotting establishment process while at the same time being essential to the inhibition of any thromboembolic event [11].

**Figure 1:**
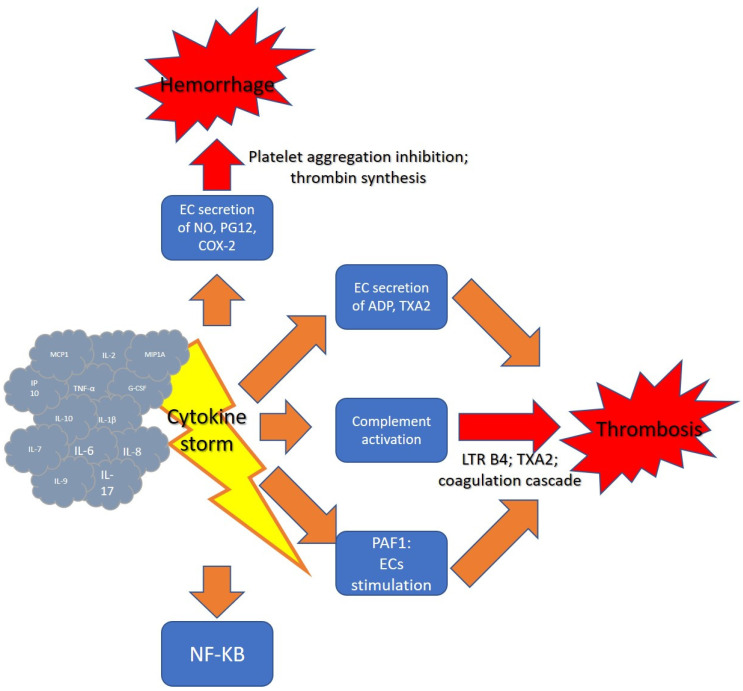
Cytokine storm and hemostasis.

In case of ECs stimulation, as after the onset of the cytokine storm, the contact of platelets to diverse factors such as thrombin, ADP and thromboxane A2 (TXA2) in the internal layer of the vessel would provoke platelet aggregation (Figure 1).

The cytokine storm can subvert the normal functions of ECs. In normal subjects, by employing the support of a surface enzyme called ecto-ATPase, the ECs cause the degradation of adenosine triphosphate (ATP) and ADP, and upon the reduced concentrations of ADP, this molecule can no longer contribute to the platelet activation procedure [12]. Moreover, normal ECs induce vasodilation and block platelet aggregation via the generation and delivery of nitric oxide (NO) and prostaglandin I2 (PGI2). NO operates as a vasodilator and a controller of thrombosis and can decrease inflammation via the inhibition of proinflammatory cytokines production and specific cellular adhesion molecules. Moreover, NO could reduce platelet adhesion and aggregation, and reduce neutrophil aggregation [13]. Contrarywise, a dysregulated activity of ECS caused by a cytokine storm can provoke an abnormal production of ADP, which is able to cause an increased platelet stimulation and thrombosis.

Furthermore, the hyperinflammation in sepsis, or other conditions able to provoke a cytokine storm, causes a systemic activation of the complement system and the generation of complement components, such as the proinflammatory peptide C5a which triggers the coagulation cascade. C5a interrelates with its receptor in neutrophils and produces powerful chemoattractant substances such as leukotriene B4, and also determines platelet aggregation through generation of thromboxane A2. The disproportionate proinflammatory mediators induce vasodilation and also block the anti-coagulant activity and fibrinolysis. This provokes microvascular congestion and the stimulation of the coagulation cascade, which determines systemic multiorgan ischemia. Severe sepsis is also associated with augmented concentrations of plasminogen activator inhibitor-1 (PAI-1) in plasma [14,15].

Finally, a novel mediator of coagulation changes in cytokine storm patients has recently been recognized. Nuclear factor (NF)-κB is a family of transcription factors that was initially found in B cells but was then promptly detected to be all overexpressed. NF-κB is responsible for the production of several proinflammatory cytokines implicated in the cytokine storm. It was observed that most of the transcriptionally regulated genes activated in ECs in response to inflammatory components such as LPS, IL-1 or TNF-α included NF-κB binding sites in their promoter regions. Later, reporter gene analyses performed employing deletion and substitution mutants demonstrated that NF-κB is of functional significance for the expression of these proinflammatory genes. Moreover, the pharmacological or genetic inhibition of NF-κB by increased stabilized IκBα mutants caused an extremely effective inhibition of EC stimulation. Moreover, NF-κB augments the production of PAF-1, which itself could enhance the probability of clot formation [16]. Other than PAF-1 and inflammation, NF-κB appears to be an essential target for the inhibition of angiogenesis [17].

Some interesting studies have been conducted on the possibility of intervening on coagulation disorders and preventing or slowing down the formation of the cytokine storm. The alteration of T cell functions, which heralds cytokine storm onset, can contribute to the coagulation changes. Cytokine production can be modified via the targeting of substances that can regulate T cell responses. Influencing T cell activities by targeting the PI3K/Akt/mTOR pathway could control the development of the cytokine storm in COVID-19 patients, while the T cell effector function can be enhanced by increasing the antiviral exhaustion through inhibiting PI3K and Akt and by targeting the downstream mTOR. Altering this signaling pathway also has the capacity to prevent the formation of thrombi, due to its action in platelet activation [18].

Finally, it is well known that the CS can cause a worse prognosis in geriatric patients. In older people, we must consider the specific effect of a proinflammatory condition on coagulation because of the peculiar state of this typology of subjects [19]. The elderly population is more vulnerable to the occurrence of both a hyperinflammatory state, such as the cytokine storm, and of critical coagulopathy, including thromboembolism. It is well ascertained that the systemic inflammatory conditions of elderly people and coagulation alteration are strictly connected—a situation which was referred to as “coagul-aging”. Physiological aging is correlated with the augmented plasma concentrations of several proteins of blood coagulation, together with fibrinolysis impairment; thus, this may be of great concern in relation to the effects of the cytokine storm in elderly patients [20].

### 2.3. COVID-19

Severe acute respiratory syndrome coronavirus 2 (SARS-CoV-2, previously 2019-nCoV) was registered in the Wuhan city of China for the first time, and since then 445,188 deaths related to the disease have been reported. It is an enveloped, positive-sense single-stranded genomic RNA virus (+ssRNA) [21,22], and entry of viral RNA into host cells occurs due to the affinity of the SARS-CoV protein S for the ACE-2 receptor [23]. The common symptoms observed in patients with COVID-19 are fever, cough, severe headache, myalgia and fatigue [24]. In most cases, SARS-CoV-2 infections occur asymptomatically or cause only mild and less fatal symptoms than MERS-CoV and SARS-CoV infections. However, in 10–20% of cases they can progress to interstitial pneumonia and acute respiratory distress syndrome (ARDS), especially in those with advanced age and associated comorbidities [25]. Since the COVID-19 pandemic first began in December 2019, SARS-CoV-2 has continuously evolved, with many variants emerging around the world [26]. These mutations, especially when they occur on the S gene encoding the spike protein (S), can affect both the viral entry into the target cells and the effectiveness of the antibodies [27]. The result is a greater visual transmissibility and a greater ability to escape the previous immunity [28]. The variants that attract the most attention as potentially dangerous to public health include B.1.1.7 (UK), P.1 (Brazilian) and B.1.351 (South African). Added to these are also variants of interest that are emerging and expanding in some countries, but are found sporadically in others, such as B.1.427 and B.1.429 (Californians) or B.1.617 (Indian) [29].

The cytokine storm caused by COVID-19 has been suggested to be associated with COVID-19 severity [30,31]. The cytokine storm is considered to be the main cause of the disease severity and death in COVID-19 patients, and is related to the high levels of circulating cytokines, severe lymphopenia, thrombosis and massive mononuclear cell infiltration in multiple organs [32]. The excessive local release of cytokines is considered to be the determinant of pathological alterations and the clinical manifestation of acute respiratory distress syndrome (ARDS).

SARS-CoV-2 infection triggers an immune response producing inflammatory cytokines along with a weak response to interferon (IFN). Thereafter, membrane-bound immune receptors and downstream signaling pathways mediate the proinflammatory immune responses of pathogenic Th1 cells and intermediate CD14 + CD16 + monocytes. This process therefore favors the infiltration of macrophages and neutrophils into the lung tissue, which leads to the cytokine storm [33].

In particular, SARS-CoV-2 can rapidly activate pathogenic Th1 cells resulting in the secretion of proinflammatory cytokines, including granulocyte-macrophage colony stimulating factor (GM-CSF) and interleukin-6 (IL-6). GM-CSF then activates inflammatory CD14 + CD16 + monocytes to produce IL-6, tumor necrosis factor-α (TNF-α) and other cytokines. Membrane-bound immune receptors, including Fc and Toll-like receptors, might contribute to an imbalanced inflammatory response, whereas weak IFN-γ induction might amplify cytokine production [34]. Neutrophil extracellular traps (NETs) might also contribute to cytokine release. Overall, the cytokine storm in COVID-19 is characterized by the high expression of IL-6 and TNF-α [35]. 

### 2.4. SARS

In 2002/2003, there was an epidemic of severe respiratory disease known as severe acute respiratory syndrome (SARS) which infected 8096 people worldwide and killed 774 (9.5%) of them. This pandemic was first reported in Guangdong Province, China, in November 2002, and spread to 29 countries around the world [36,37]. SARS-CoV is a positive, single-stranded, enveloped RNA virus. Its RNA encodes a nonstructural polyprotein replicase and structural proteins, including spike (S), envelope (E), membrane (M) and nucleocapsid (N) proteins, which causes infection first from the upper respiratory tract to affect the lower one, characterized by functionally critical lung damage through the binding of the spike protein with ACE2 and the subsequent downregulation of this receptor [38,39]. Lymphopenia and thrombocytopenia are commonly present. The decrease of both CD4+ and CD8+ T-lymphocytes occurs early and adversely affects the prognosis [40]. The animal reservoir of SARS-CoV in nature remains to be identified.

During SARS-CoV infection, the levels of IL-1β, IL-6, IL-8, IL-12, inducible protein 10 (IP-10), MCP-1 and IFN-γ increase significantly [41]. This is countered by low levels of the cytokine Th2 IL-4 [42].

SARS and COVID-19 infections can cause MAS and cytokine storms. Massive release of proinflammatory mediators, including IL-6 and IL-1, contributes to vascular permeability, plasma loss and DIC processes, thus causing lung damage and ARDS, as well as multi-organ failure [43,44]. In SARS-Cov and MERS-Cov, increased IFN-γ levels have been associated with inflammation and extensive lung damage [45,46]. The elevated levels of IL-6 in the pathogenesis of SARS-Cov-1 lays the foundation for the hypothesis that the two members of the Coronaviridae family may indeed share common pathophysiological mechanisms [47]. High viral concentrations and dysregulated cytokine / chemokine responses cause a "cytokine storm" with lung immunopathological changes following SARS-CoV infection [48].

An interferon-related cytokine storm was induced by post-SARS coronavirus infection, thus contributing to the genesis of immunopathological damage in SARS patients [49].

### 2.5. MERS

Coronavirus Middle East respiratory syndrome (MERS-CoV), a positive-sense single-stranded RNA (ssRNA) genome about 30 kb in size [50], develops in Saudi Arabia in June 2012, and as of 16 October 2018, over 2260 cases of confirmed MERS-CoV infection and 803 related deaths have been reported [51]. MERS-CoV uses the N-terminal part of its peak, the so-called S1 protein, to bind to two host cell surface molecules, dipeptidyl peptidase-4 (DPP4) and α2,3-sialic acids [52]. The major clinical manifestations are fever, chills, cough, shortness of breath, generalized myalgia, malaise, drowsiness, diarrhea, confusion, dyspnea and pneumonia [53]. Although in most subjects the disease progresses asymptomatically or paucisymptomatically, in patients with comorbidities such as diabetes, renal insufficiency and underlying immunosuppression, the clinical manifestations are markedly more serious and potentially fatal [54].

Middle East respiratory syndrome coronavirus (MERS-CoV) became one of the most serious pandemics of last 30 years. As with the other pandemic, it was also clinically characterized by ARDS, and in turn associated to a significant cytokine storm occurrence [55].

More specifically, MERS-CoV infection was reported to induce increased concentrations of IL-15, IL-17, IFN-γ and TNF-α [36,45].

In the past, significantly higher serum levels of proinflammatory cytokines (IL-6, IFN-α), and chemokines (IL-8, CCL5, CXCL8 and CXCL-10) were also detected in patients with severe SARS-CoV or MERS-CoV infections compared to those with milder infections [56,57]. Upregulation of proinflammatory cytokines, notably the IL-6, together with downregulation of antiviral cytokine, was observed in MERS-CoV infections [44,58].

### 2.6. H1N1 Influenza A

On 21 April 2009, the Centers for Disease Control and Prevention (CDC) have confirmed two cases of febrile respiratory disease caused by an infection with a new influenza A (H1N1) virus in pediatric age in Southern California [59]. The pandemic (H1N1) influenza A virus is a novel reassortant virus comprising two swine strains, one human strain and one avian strain of influenza [60]. Clinically, the infection occurs frequently with the onset of symptoms such as fever, cough, sore throat, runny nose, body ache, headache, chills and fatigue. A significant number also have gastrointestinal symptoms, such as diarrhea and vomiting. In the most severe forms, the chest X-ray shows an image of pneumonia and mild interstitial fibrosis [61].

Influenza A viruses proliferate in human epithelial cells, which produce inflammatory cytokines/chemokines as a “cytokine storm”, eventually attenuated with the viral nonstructural protein 1 (NS1) [62]. Notably, the uncontrolled viral replication and the associated “cytokine storm” of IL-6, IL-8, IP-10, MIG and MCP-1 are responsible for this infection’s serious clinical manifestations and poor outcomes [63,64,65].

In the presence of the H_2_O_2_-MPO system, viral NS1 protein produced in the cells is associated with the enhanced production of large amounts of the chemokines IL-8 by neutrophils and MCP-1 by macrophages, suggesting that NS1 of the H1N1 (PR-8) influenza virus may play a key role in the “cytokine storm” when the H_2_O_2_-MPO system is active [66].

In animal models, the studies exclude a role for lymphocytes as key regulators of the influenza virus-induced cytokine storm [67]. S1P_1_ receptor signaling in lung endothelial cells suppresses the cytokine storm. The infiltration of macrophages and NK cells alone does not appear to be associated with cytokine storms.

Chemokines such as CCL2, CCL3, CXCL2 and CXCL10 induce the recruitment of innate immune cells into the lungs, exacerbating the cytokine storm and further damaging the lungs. Overall, patients suffering from H1N1-induced pneumonia and consequent ARDS have excessively elevated levels of serum interferons, cytokines and chemokines, which is characteristic of a cytokine storm [68,69,70]. Although the immune dysregulation observed in these individuals varies with the severity of the disease; it has been observed that some mediators, more than others, are more commonly reported, as in the case of IFN-γ, IL-6, IL-1α, IL-1β, TNF-α, IL-15, IL-12p70, IL -17, IL-10, MCP-1, MIP-1β, IL-8, MIG, IP-10, MIP-1α, GM-CSF and RANTES [71,72,73,74,75].

In addition, a positive association was found between IL-6 levels and disease severity [76,77,78,79].

### 2.7. Spanish Flu

The Spanish flu first developed in the United States in the spring of 1918 [80]. The virus that supported the pandemic was a type A strain, subtype H1N1, which causes upper and lower respiratory tract infections in the infected host, resulting in symptoms such as nasal discharge, chills, fever, decreased appetite and possible lower respiratory tract disease [81,82]. Data available to us report how the Spanish flu infected about 500 million people worldwide, causing the deaths of 50–100 million people (3% to 5% of the world population), thereby making it one of the deadliest pandemics in human history [83].

From the structural point of view, the viruses belonging to the type A strain are subtyped on the basis of their combination of surface glycoproteins NA and HA, whose binding to the receptor glycans with terminal sialic acids in different configurations with underlying sugars is responsible for the attachment to respiratory epithelial cells [84,85]. Currently 18 HA and 11 NA subtypes are known, which mainly circulate in water wild birds, which are considered the natural reservoir for this type of virus [86].

The 1918–1919 influenza pandemic mortality curve shows a “W” shape with higher mortality in young adults aged 20 to 40, probably due to the lack of pre-existing immunity in this population. It is possible that an antigenically similar strain of influenza circulated before 1889, providing a level of protection against the new pandemic strain H1N1 [87].

Data on the Spanish flu and its associated cytokine storm are few and somewhat anecdotal. Current evidence suggests that viral factors, including haemagglutinin and polymerase gene segments, probably contributed to a massive, dysregulated proinflammatory cytokine storm in victims of the pandemic [88]. This hypothesis is based upon experimental studies carried out in a number of animal models through the reconstructed 1918 influenza virus. Such research showed that the 1918 influenza virus triggered a massive, dysregulated proinflammatory response, likely to contribute to severe lung lesions [89,90,91,92]. Such dysregulated immune response has also been observed in both the avian H5N1 virus and the 2009 pandemic influenza virus [93,94].

Furthermore, a recent study in ferrets proved that the 1918 influenza virus could spread to and induce cytokine responses in tissues outside of the respiratory tract; this fact likely contributed to the severity of the infection [95,96] and could explain the neurological complications observed during the Spanish flu outbreak [97,98].

By analyzing the different immunopathological processes that characterize the emerging and re-emerging respiratory viral diseases that have had a global impact in human history (Spanish flu, SARS, MERS, influenza A H1N1 2009 and COVID-19), the hyperinflammation known as “cytokine storm syndrome (CSS)” appears as the connecting key between them. In Table 1 we have reported the main cytokines and chemokines involved in the various respiratory viral pandemics.

**Figure 2:**
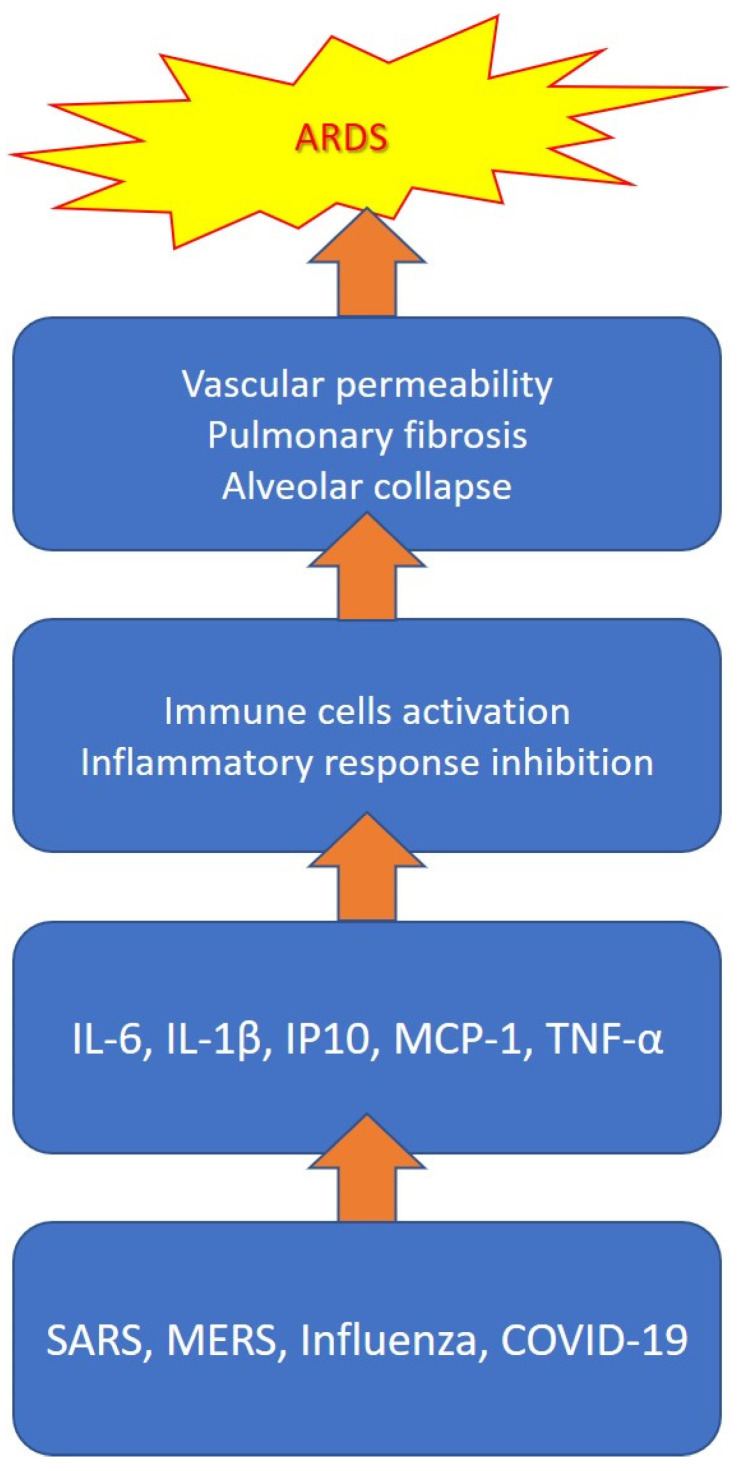
Cytokine storm during viral infection.

Although it is not yet clear what the determinants of the systemic inflammatory response associated with a high cytokine release are, it is believed that it is caused by an imbalance in the regulation of the immune system (the increased activation of immune cells, decreased anti-inflammatory response, etc.) [99]. The massive and continuous release of proinflammatory cytokines and chemokines is responsible for life-threatening severe acute respiratory failure, in addition to pulmonary fibrosis and alveolar collapse processes with long-term negative outcomes [100]. In particular, IL-1β, IL-6 and TNF-α turned out to be the mediators most involved in the genesis of severe respiratory forms, as they play a central role in determining diffuse tissue damage [101]. In Figure 2, we have summarized the main mediators involved in the cytokine storm and their pathogenetic role in the development of ARDS during viral infection.

The effects exerted by the cytokine storm on the processes of hemostasis are implemented through the action on different targets, such as endothelial cells and platelets, and the mediation of multiple effectors, such as nitric oxide, complement, adenosine diphosphate (ADP) and NF-kB. In fact, recent studies have shown that some cytokines and chemokines involved in CCs, including interleukin-6 (IL-6), interleukin-1β (IL-1β), induced protein 10 (IP10) and chemotactic protein-1 of monocytes (MCP-1), were significantly elevated in severe SARS, MERS and COVID-19 [102].

**Figure 3:**
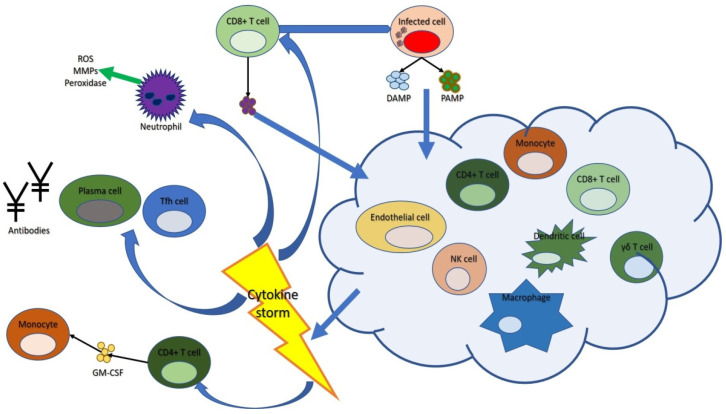
Viral dissemination during development of cytokine storm.

It also emerged that level of IL-6 and C reactive protein are considered useful indicators capable of predicting severe respiratory forms, and thus allowing to correctly identify the patients who may benefit from an early escalation of the treatment [103]. IL-6, in fact, plays a crucial role in the pathogenesis of CSS by increasing vascular permeability with interstitial edema, causing activation of the complement system and favoring the release of histamine from mast cells. It also activates the coagulation system with consequent procoagulation effects, up to disseminated intravascular coagulation (DIC) [104,105]. Polidoro RB et al. showed that, following the development of the cytokine storm during Sars-Cov infection, the release of C-reactive protein (CRP) and serum amyloid A (SAA) occurs, which typically increase in the first days if the patient develops moderate or severe symptoms. These levels may decrease after 7 days as haptoglobin and fibrinogen increase, possibly resulting in thrombi formation in the pulmonary circulation, which can lead to the development of severe respiratory syndrome [106]. However, the experimental scientific evidence also suggests the potential negative consequences deriving from an increase in IL-6 levels on the cellular immune response against viruses, and in particular on viral clearance, favoring the establishment of a persistent viral state in the infected hosts [107]. Recent studies have shown that cytokine storms (in particular, elevated levels of IL2R, IL-6 and TNFα), along with other conditions, such as advanced age, immunocompromise and corticosteroid therapy, are responsible for a prolonged loss of viral RNA in patients with COVID-19 [108,109]. Figure 3 shows how the cytokine storm may favor viral dissemination.

## 3. Conclusions

Considering the crucial role of proinflammatory pathogenetic mechanisms in severe viral infections affecting the respiratory tract, we believe that a greater knowledge of these processes could allow us to identify, not only the most suitable treatment, but also the strategies to be prepared for the next viral pandemic. In consideration of this, immunomodulatory therapy can therefore be a beneficial addition to antiviral therapy. In fact, anti-IL6 monoclonal antibodies tocilizumab (already approved for the treatment of CAR T cell-induced cytokine release syndrome), siltuximab and sarilumab are currently being studied in the management of severe COVID-19 [110]. More recently, studies have been conducted that focus on inhibiting IL-1β to counteract the cytokine storm using Anakinra, an IL-1β antagonist, which has been shown to radically improve the survival rates of patients with severe sepsis [111]. IL-1 exerts chemotactic actions on immune cells, inducing the production of secondary cytokines with consequent acute phase reactions [112,113]. Cytokine storms could also be considered as a potential factor responsible for the development of neuropathies after severe infection, contributing to the development of chronic pain after the acute COVID-19 infection resolves [114]. However, not all patients with respiratory viruses develop a cytokine storm, and therefore the clinically severe forms of the disease. The reason is probably to be found in the individual genetic susceptibility, which predisposes some subjects to this complication. A recent study analyzed 671 severe cases of non-COVID-19 bacterial and viral pneumonia and 2910 nonserious cases of bacterial and viral pneumonia by identifying a significant association of the IL-6-174C allele (associated with a level of IL-6 plus elevated) with the severity of pneumonia. Likewise, the genes of the interferon type 1 (IFN1) pathway, and the coding ones of the activating receptor of the natural killer cells (NKG2C), have also been identified as potentially involved in antiviral responses in the lungs [115].

Identifying the diagnostic strategies and the proinflammatory markers capable of predicting the evolution to clinically severe forms is of crucial importance in the early management of patients, and in increasing their survival.

It is necessary to analyze our past, not in order to obtain easy answers and equally easy recipes, but with the aim of projecting it into the vision of a probable or plausible future.

## Figures and Tables

**Table 1:** Involvement of main cytokines in viral pandemics.

Cytokine	Source	Function	Effects in Viral Pandemics
IL-1β	Macrophages, monocytes	Proinflammation, proliferation, apoptosis, differentiation	Increase vascular permeability, pulmonar fibrosis, alveolar collapse
IL-6	Macrophages, T-cells, adipocyte	Proinflammation, differentiation, cytokine production	Diffuse tissue damage, genesis of severe respiratory forms
CXCL10	Leukocytes, neutrophils, eosinophils, monocytes, endhotelial cells	Chemotaxis, apoptosis, cell growth inhibition, angiostasis	Recruitment of innate immune cells into the lung with consequent tissue damage
MCP-1	Macrophages, fibroblasts	Migration and infiltration of monocytes/macrophages	Responsible for infection serious clinical manifestations and poor outcomes
TNFα	Macrophages, NK cells, CD4^+^ lymphocytes, adipocyte	Proinflammation, cytokine production, cell proliferation, apoptosis	Increased perivascular infiltration of activated macrophages, neutrophils and fibroblasts, fibrin deposition and alveolar collapse, leading to acute respiratory distress syndrome (ARDS)
CXCL8	Macrophages, air way smooth muscle cells, endothelial cells	Chemotactic action for neutrophils and monocytes in the respiratory tract	Involvment in the inflammation and trafficking of immune cells in the context of viral infections
